# Rhizosphere Microbial Communities and Geochemical Constraining Mechanism of Antimony Mine Waste-Adapted Plants in Southwestern China

**DOI:** 10.3390/microorganisms10081507

**Published:** 2022-07-26

**Authors:** Xiaofeng Xie, Shangyi Gu, Likai Hao, Tianyi Zhang, Zidong Guo

**Affiliations:** 1 College of Resources and Environmental Engineering, Guizhou University, Guiyang 550025, China; xiexf2016@163.com (X.X.); zhangtianyi1995@126.com (T.Z.); guozd0502@163.com (Z.G.); 2 Key Laboratory of Karst Geological Resources and Environment, Ministry of Education, Guiyang 550025, China; 3 State Key Laboratory of Environmental Geochemistry, Chinese Academy of Sciences, Guiyang 550002, China

**Keywords:** antimony, Qinglong, rhizosphere microorganisms, slag, tailings, waste rock

## Abstract

Antimony (Sb) and arsenic (As) are two hazardous metalloid elements, and the biogeochemical cycle of Sb and As can be better understood by studying plant rhizosphere microorganisms associated with Sb mine waste. In the current study, samples of three types of mine waste—Sb mine tailing, waste rocks, and smelting slag—and associated rhizosphere microorganisms of adapted plants were collected from Qinglong Sb mine, southwest China. 16S rRNA was sequenced and used to study the composition of the mine waste microbial community. The most abundant phylum in all samples was *Proteobacteria*, followed by *Bacteroidota*, *Acidobacteriota*, and *Actinobacteriota*. The community composition varied among different mine waste types. *Gammaproteobacteria* was the most abundant microorganism in tailings, *Actinobacteria* was mainly distributed in waste rock, and *Saccharimonadia*, *Acidobacteriae*, and *Ktedonobacteria* were mainly present in slag. At the family level, the vast majority of *Hydrogenophilaceae* were found in tailings, *Ktedonobacteraceae*, *Chthoniobacteraceae*, and *Acidobacteriaceae* (Subgroup 1) were mostly found in slag, and *Pseudomonadaceae* and *Micrococcaceae* were mainly found in waste rock. *Actinobacteriota* and *Arthrobacter* are important taxa for reducing heavy metal(loid) mobility, vegetation restoration, and self-sustaining ecosystem construction on antimony mine waste. The high concentrations of Sb and As reduce microbial diversity.

## 1. Introduction

Antimony (Sb) is a potentially toxic and carcinogenic metalloid element. Humans and animals can be exposed to Sb in the environment via water, air, food, skin contact, and respiration. Long-term skin contact with dust containing Sb can lead to Sb spots, and inhalation of low concentrations of Sb dust or Sb-containing fumes can induce pneumoconiosis, lung cancer, and other diseases [1,2,3]. Sb and its compounds are also listed as priority pollutants by the United States Environmental Protection Agency and the European Union [4,5,6].

Mining activity is a major cause of the release of anthropogenic Sb into the environment [6]. Mining, flotation, and smelting are indispensable components of the process of mineral production, and they generate large amounts of mining waste such as mining waste rock, tailings, and smelting slag. Low metal recovery in some mines leads to mine waste that is potentially risky to humans and the environment due to its high heavy metal(loid) content [7,8]. In addition, many mine wastes are directly disposed of in the mine site without any treatment, which further increases the risk of heavy metal(loid) pollution [9]. China’s Sb ore reserves and production are the largest in the world, and Sb mining and smelting are the main source of Sb pollution, which may cause Sb levels in the atmosphere, water, and soil to exceed the standard [4].

The Sb content in the soil around the Xikuangshan Sb mine in Hunan ranged from 527 to 11,798 mg/kg [10]. Fu et al. [11] reported that the Sb content in water around Xikuangshan ranged from 5.6 to 163 μg/L (mean 24.7 μg/L), and in soils it ranged from 141 to 8733 mg/kg (mean 1315 mg/kg). High Sb levels were also detected in tailings (68.0–417,196 mg/kg, mean 3789 mg/kg), fish (1.0–1112 μg/kg, mean 86.8 μg/kg), surrounding plants (0.1–609 mg/kg, mean 13.5 mg/kg), and vegetables in water (0.1–10.7 mg/kg, mean 2.3 mg/kg). Liu et al. [12] reported that Sb levels in the hair of residents from Xikuangshan Sb mine (0.250–82.4 mg/kg, mean 15.9 mg/kg) and Qinglong Sb mine (0.060–45.9 mg/kg, mean value 5.15 mg/kg) were significantly higher than those of residents of from Guiyang City (0.065–2.87 mg/kg, mean 0.532 mg/kg).

The establishment of vegetation caps on mine wastes to reduce the mobility of heavy metals is considered an effective method to mitigate mining waste contamination. Plant covering also increases organic matter content, cation exchange capacity, and nutrient levels, further improving the chemical and biological properties of contaminated soils, thus creating a self-sustaining ecosystem [13,14]. However, plant growth in mining waste sites is frequently constrained by harsh field conditions, which are characterized by low nutrient and organic material contents, high levels of heavy metals, and/or low pH.

Rhizosphere microorganisms have a strong influence on plant growth and survival, and synergistic interactions between plants and rhizosphere microorganisms facilitate the removal of heavy metals from soil [15]. Rhizosphere microbes are also considered an important pathway for nutrient uptake by plants, directly affecting plant productivity and soil ecosystem function [16]. Plant rhizosphere bacteria can mitigate the adverse effects of nutrient deficiencies and metal(loid) pollution, promote plant growth in the soil [17], fix atmospheric nitrogen, and dissolve minerals such as phosphate, providing essential nutrients to plants [15,18,19]. Rhizosphere microorganisms can immobilize heavy metals at the rhizosphere level, and can also influence the uptake of heavy metals by plants by affecting their speciation in the soil [20]. Functional groups on microbial cell walls, including hydroxyl, carboxyl, sulfhydryl, and amino groups, can adsorb metal ions [21]. Microorganisms can mediate the transformation of Sb and arsenic (As) in soil–plant systems, thereby modifying the toxicity and mobility of As and Sb [22]. For example, Sb-oxidizing bacteria can rapidly oxidize Sb(III) to Sb(V) and reduce the transport capacity of Sb, thus reducing Sb uptake by plants [23,24,25]. *Shewanella oneidensis* can immobilize Sb through adsorption and complexation [26]. In addition to bacteria, as a key component of soil microbial communities, fungi can release nutrients from decomposed dead organisms, and drive material cycles. As the main decomposer and carbon sequester in soil, fungi can maintain soil fertility and health, and play an important ecological role in the ecosystem [27,28,29]. Jia et al. [30] found that arbuscular mycorrhizal fungi changed the oxidation of heavy metals and bacterial community structure in rhizosphere soil, reducing the bioavailability of heavy metals.

The synergistic evolution of plants and rhizosphere microorganisms in harsh environments makes the use of rhizosphere microbial composition a prerequisite for the remediation of heavy metal-contaminated vegetation at mine waste sites [31]. Soil properties are an important determinant of plant rhizosphere microbial community composition [32]. Microbial communities and their metabolic activities are also affected by extreme geochemical conditions [33]. Environmental factors such as nutrient elements, pH, temperature, and especially heavy metal content can control the distribution and abundance of microorganisms. Mining wastes (including tailing, smelting slag, and mining waste rock) with high metal(loid) content have important inhibitory effects on microorganisms (including fungi and bacteria) [34,35,36,37,38], and some studies [39] have shown that antimony and arsenic have stronger inhibitory effects on soil bacteria than fungi. Therefore, this study focuses on rhizosphere bacteria.

Microbial community composition under heavy metal contamination conditions has become one of the hotspots of current research, but very little research has been conducted on the rhizosphere microorganisms associated with Sb mine waste-adapted plants. Only a few studies have been conducted on Sb mine tailing microorganisms [31,40,41]. Even less research has investigated plant rhizosphere microorganisms adapted to smelting slag and mining waste rocks. It is critical to investigate the microbial composition of mine waste-adapted plants’ rhizospheres in order to understand the biogeochemical cycle process of heavy metal(loid)s, and to prevent and control heavy metal(loid)s.

In the current study, we characterized the rhizosphere microbial communities of native adapted plants from three types of mining waste from the Qinglong Sb mine in southwestern Guizhou, China: tailing, smelting slag, and mining waste rocks. High-throughput sequencing was used to characterize the rhizosphere microbial communities. The objectives of the study were to (1) understand Sb and As contamination in the three types of mine waste and the compositions of plant rhizosphere microbial communities, (2) compare plant rhizosphere microbial community compositions and diversity in the three waste types, and (3) investigate the underlying mechanisms responsible for differences in microbial community diversity in the three waste types.

## 2. Materials and Methods

### 2.1. Site Description and Sampling

**Figure 1:**
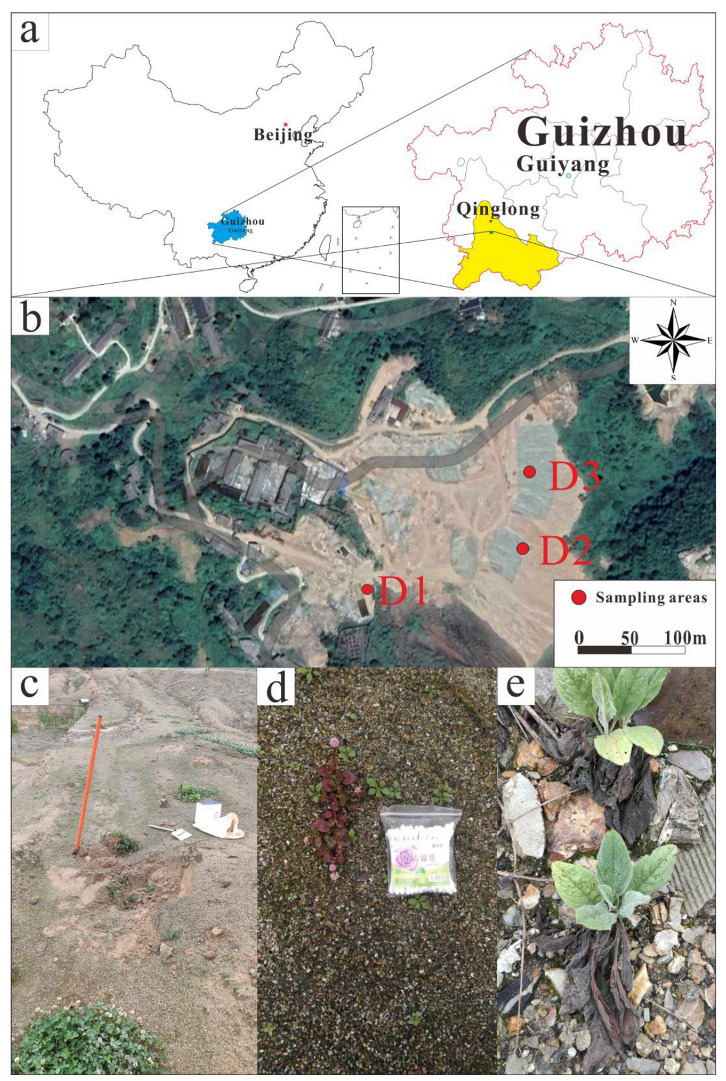
(**a**): Geographical location map of Qinglong Antimony Mine; (**b**): Distribution map of sampling points, where D1 is the tailing area, D2 is the slag area, and D3 is the waste rock area; (**c**): Tailing and *Trifolium repens* L.; (**d**): Slag and *Polygonum capitatum*; (**e**): Waste rock and *Cynoglossumlanceolatum* Forssk.

The Qinglong Sb mine is located in Qinglong County in the southwest of Guizhou Province, China (25°40′22″ N, 105°10′25″ E). The area is a typical karst landscape with abundant underground water resources. Qinglong has a mild subtropical monsoonal climate with an average annual temperature of 14 °C and average annual rainfall of 1380 mm. It is estimated that 2,883,700 t of mine waste is piled up in the Qinglong Sb mine area, including 2,700,600 t of mining waste rock, 154,600 t of smelting slag, and 28,500 t of flotation tailings [42]. The sampling location map is shown in Figure 1a,b. The tailing is silt-like, earthy yellow, and fine-grained (Figure 1c). The slag is unevenly sized and brown. Large slag has developed pores, and iron (Fe) oxides can be visibly seen on the surface. Quartz, gypsum, kaolinite, and Fe oxides can be seen on the fine slag (Figure 1d). The waste rock is mostly composed of ore body surrounding rocks and intercalated rocks, in blocks of different sizes. The main minerals are quartz, fluorite, limonite, and calcite (Figure 1e).

Herbaceous and woody plants have been developed to varying degrees in the mine area. In the mine area, we found differences in the types of plants associated with different types of mine wastes. Only *Polygonum capitatum* Buch.-Ham. ex D. Don Prodr was found in the slag area, whereas four plants, namely, *Trifolium repens* L., *Rumex acetosa* L., *Plantago asiatica* L., and *Conyza canadensis* (L.) Cronq. were growing on both tailings and waste rock. In addition to the above plants, *Cynoglossum lanceolatum* Forssk., *Brassica juncea* (L.) Czern. et Coss., and *Raphanus sativus* L. were also found in the waste rock area. Samples were collected in April 2021, and eight species of adapted plants from the mining area were selected; *T. repens* L. (n = 8), *R. acetosa* L. (n = 8), *P. asiatica* L. (n = 8), *C. canadensis* (L.) Cronq. (n = 8), *P. capitatum* Buch.-Ham. ex D. Don Prodr (n = 6), *C. lanceolatum* Forssk. (n = 4), *B. juncea* (L.) Czern. et Coss. (n = 1), and *R. sativus* L. (n = 1).

For each plant, the roots were dug out with a spade and the excess soil was shaken off, and approximately 200 g of rhizospheric soil samples was taken for chemical analysis. For analysis of the microbial composition, rhizosphere soil samples attached to the root surface were deposited into 1.5 mL microcentrifuge tubes using sterile cotton swabs. Samples were transported back to the laboratory on ice (4 °C), then stored at −40 °C prior to DNA extraction. A total of 44 soil rhizosphere microbial samples were collected, with each plant corresponding to one rhizosphere microbial sample (Table 1).

### 2.2. Chemical Analysis

The samples for chemical analysis were naturally air-dried, then the plant roots, leaves, and gravel were manually removed and passed through a 2 mm sieve. The samples were then ground in an agate mortar and passed through a 200-mesh sieve.

#### 2.2.1. pH Analysis

For pH determination, 10 g of sample was passed through a 2 mm nylon sieve into a centrifuge tube, 25 mL of ultrapure water was added at a soil-to-water ratio of 1.0:2.5, then the preparation was shaken for 20 min at room temperature to allow the soil to disperse sufficiently. After resting the preparation for 30 min, pH was measured using a calibrated Five Easy Plus PE 28 pH meter (Mettler Toledo, Shanghai, China). pH tests were conducted at the Key Laboratory of Karst Geological Resources and Environment (Guiyang, China).

#### 2.2.2. Sb and As Analysis

The sieved samples were digested by heating with concentrated HNO_3_ and HCl (1:3 *v*/*v*) for 2 h at 100 °C. An appropriate amount of supernatant was added to 2.5 mL of a 5% thiourea-ascorbic acid mixture which was then incubated at room temperature for 30 min for pre-reduction. Total Sb and As concentrations were then determined via hydride generation-atomic fluorescence spectrometry (HG-AFS 8510, Beijing Haiguang Instrument Co., Beijing, China). The operating conditions of AFS were optimized, and the calibration curves of Sb and As exhibited good linearity (r > 0.999). Sb and As tests were conducted at the Key Laboratory of Karst Geological Resources and Environment (Guiyang, China). The standard reference material GBW07985 (Chinese National Standard) was used for quality control.

#### 2.2.3. Detection of Major Elements

Samples were dried at 105 °C and placed in a platinum crucible, and a mixed melt of lithium tetraborate-lithium metaborate-lithium nitrate was added and mixed thoroughly, and then melted at a high temperature to make a partial flat glass sheet. The sheet was analyzed for major elements via an X-ray fluorescence spectrometer (PANalytical PW2424, Almelo, The Netherlands). Analysis of major elements (including iron, potassium, magnesium, phosphorus, and sulfur, RSD < 5%) was conducted at ALS Minerals—ALS Chemex Co. Ltd. (Guangzhou, China). The water used in the experiments was ultrapure water prepared by an ultrapure water instrument (UPTplus-20, Shanghai Lichen-BX Instrument technology Co., Shanghai, China, Resistivity > 18.2 MΩ·cm), the reagents were of superior purity or above, and the experimental vessels were soaked in 10% nitric acid solution for more than 24 h in advance and cleaned with ultrapure water.

### 2.3. High-Throughput Sequencing of the 16S rRNA V4 Region

The samples were sent to Novogene Bioinformatics Technology Co., Ltd. (Beijing, China) for DNA extraction and further sequencing. The CTAB method was used to extract total genomic DNA from samples [43]. DNA concentration and purity were monitored via 1% agarose gels. DNA was diluted to 1 g/L in sterile water [44]. The 515f/806r primer pair was used to amplify V4 hypervariable regions of 16S rRNA genes (515f: 5′-GTGYCAGCMGCCGGTAA-3′; 806r: 5′-GGACTACHVGGGTWTCTAAT-3′) [45]. All PCR reactions were conducted with 15 μL of Phusion^®^ High-Fidelity PCR Master Mix (New England Biolabs, Ipswich, MA, USA), 0.2 μM of forward and reverse primers, and approximately 10 ng of template DNA. Thermal cycling consisted of an initial denaturation at 98 °C for 1 min, followed by 30 cycles of denaturation at 98 °C for 10 s, annealing at 50 °C for 30 s, and elongation at 72 °C for 30 s, then a final incubation at 72 °C for 5 min. PCR products were mixed with the same volume of IX loading buffer containing SYB green, then electrophoresed on 2% agarose gels for detection. PCR products were mixed at equidense ratios, then the mixture of PCR products was purified with the Qiagen Gel Extraction Kit (Qiagen, Hilden, Germany). Sequencing libraries were generated using the TruSeq^®^ DNA PCR-Free Sample Preparation Kit (Illumina, San Diego, CA, USA) in accordance with the manufacturer’s recommendations, and index codes were added. Library quality was assessed with a Qubit@2.0 Fluorometer (Thermo Scientific, Waltham, MA, USA) and the Agilent Bioanalyzer 2100 system. Lastly, the library was sequenced on the Illumina NovaSeq Platform (Novogene Bioinformatics Company, Beijing, China) and 250 bp paired-end reads were generated. Paired-end reads were merged using FLASH (VI.2.7, http://ccb.jhu.edu/software/FLASH/, accessed on 10 May 2021) [46]. Quality filtering on the raw tags was performed under specific filtering conditions to obtain high-quality clean tags [47] in accordance with the QIIME (V1.9.1, http://qiime.org/scripts/split_libraries_fastq.html, accessed on 10 May 2021) [48] quality control process. The tags were compared with the reference database (Silva database, https://www.arb-silva.de/, accessed on 10 May 2021) using the UCHIME algorithm (http://www.drive5.com/usearch/manual/uchime_algo.html, accessed on 14 May 2021) [49] to detect chimera sequences, then the chimera sequences were removed [50]. Effective Tags were thus obtained.

The UPARSE algorithm (UPARSE v7.0.1001, http://www.drive5.com/uparse/, accessed on 17 May 2021) [50] was used to cluster all Effective Tags of all samples. By default, the sequence is clustered into operational statistical units (OTUs) with 97% identity. At the same time, representative sequences of OTUs are selected. In accordance with the algorithm principle, the sequence with the highest frequency of OTUs is selected as the representative sequence of OTUs. The OTU sequences were annotated, and species annotation analysis was conducted using the Moth method and the SSUrRNA database of SLILVA138, with the threshold set at 0.8–1.0 (http://www.arb-silva.de/, accessed on 23 May 2021) [51,52]. Taxonomic information was obtained and the community composition of each sample was counted at each classification level: kingdom, phylum, class, order, family, genus, and species. MUSCLE Software [53] (Version 3.8.31, http://www.drive5.com/muscle/, accessed on 23 May 2021) was used to perform fast multi-sequence alignment to obtain the system occurrence relations of all OTU representative sequences. Raw sequences obtained in this study were submitted to the NCBI Sequence Read Archive database under the accession number PRJNA849367.

### 2.4. Statistical Analysis

Qiime software (version 1.9.1) was used to calculate observed OTUs, Chao1, Shannon, Simpson, ACE, goods coverage, and PD_whole_Tree index. R software (version 2.15.3) was used to generate rarefaction curves. Weighted UniFrac distances were used to assess similarities among the microbial communities in the three sample types [45]. UniFrac distance-based principal coordinate analysis (PCoA) was used for better visualization of complex multidimensional data [54]. PCoA was performed using the R software packages WGCNA, stats, and ggplot2. Redundancy analysis conducted via CANOCO 5 (Microcomputer Power, Ithaca, NY, USA) was used to identify the major geochemical parameters that most influenced the structure of the microbial community. Redundancy analysis was performed to identify possible associations between microbial communities (limited to class with abundance in the top 10) and selected geochemical parameters. The position of symbols relative to the vector head indicates the correlation between the microbial community and the geochemical parameters. The length of the arrow reflects the relative importance of these environmental factors in distinguishing the entire microbial community within one library. Sankey maps of the 10 most abundant microorganisms at each level were generated using the Origin2021 software package. Correlations between geochemical parameters and the abundance of selected microorganisms were determined via Spearman’s correlational analysis.

## 3. Results

### 3.1. Geochemical Properties of Mine Waste

**Figure 2:**
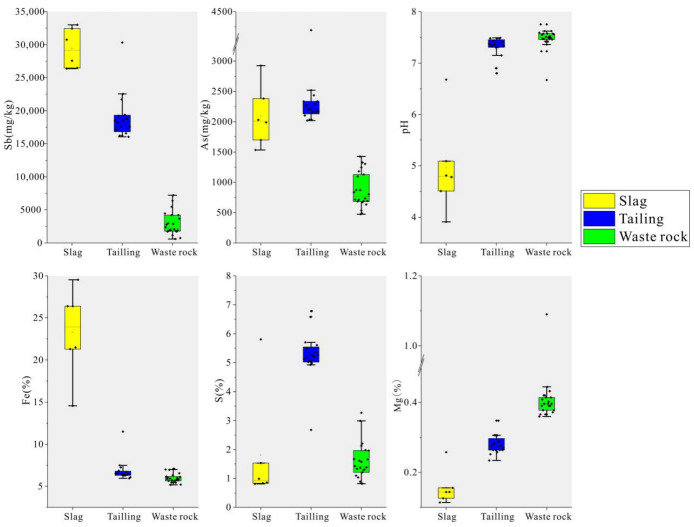
Box line diagram of geochemical parameters of three mine wastes.

The geochemical properties of the three types of mining waste are shown in Table S1. In the Qinglong Sb mining area, the hierarchy of Sb content was slag (29,438 ± 2737 mg/kg) > tailing (19,044 ± 3424 mg/kg) > waste rock (2912 ± 1731 mg/kg). The hierarchy of As content was tailing (2342 ± 498 mg/kg) > slag (2093 ± 458 mg/kg) > waste rock (870 ± 281 mg/kg). The distribution of geochemical parameters is shown in Figure 2. Tailing and waste rock were neutral to weakly alkaline overall, whereas slag was acidic with pH values ranging from 3.9 to 6.7. The total Fe content in the slag was significantly higher than that in tailing and waste rock, and the total S content in tailing was the highest. The hierarchy of Mg content was waste rock > tailing > slag.

### 3.2. High-Throughput Sequencing Analysis

**Figure 3:**
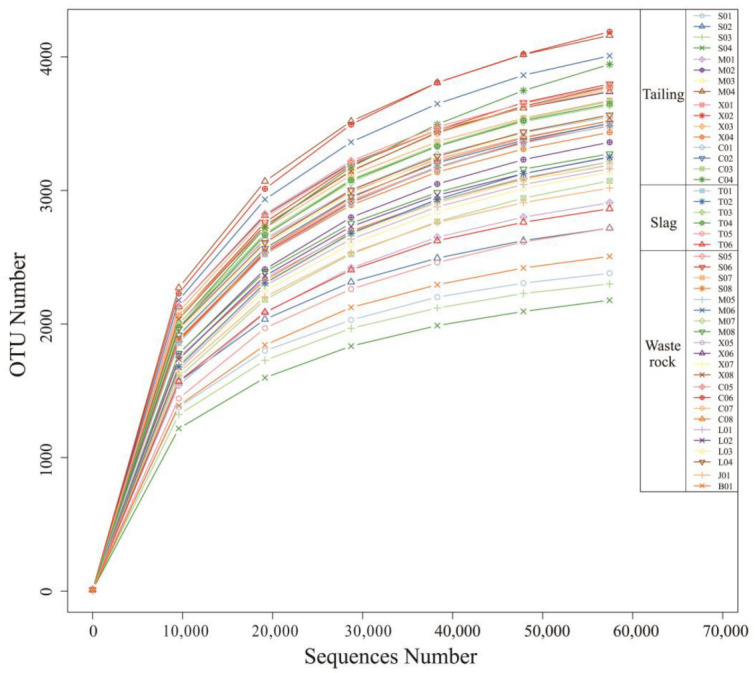
Rarefaction curves for the number of OTUs.

The Illumina NovaSeq Platform was used for splicing and quality control of bacterial sequencing data from 44 rhizosphere microbial samples of Sb mine plants in Qinglong, followed by chimera filtering, yielding 2,862,909 16S rDNA sequences with an average length of 253 nt. Effective Tags of all samples were clustered with 97% identity for OTU clustering, then the sequences of OTUs were annotated with species. Sparsity curves are shown in Figure 3. The rate of OTUs obtained from the annotation decreased as the number of sample reads increased. Samples C06 and M04 had the highest numbers of OTUs (4684 and 4601, respectively), and samples S04 and S03 had the lowest (2416 and 2555, respectively).

Alpha diversity including Observed species, Shannon, Simpson, Chao1, ACE, Goods coverage, and PD whole tree was calculated for each sample. In the alpha diversity index, community diversity was analyzed via Shannon and Simpson indices, and community richness was analyzed via Chao1 and ACE indices. Goods coverage indicates the coverage of the measured flora in all communities, and higher values represent a higher probability of sequences being detected in the samples. The Shannon index was positively correlated with microbial diversity, and the larger the index value, the higher the microbial diversity in the sample. Simpson’s Index of Diversity 1-D was also positively correlated with microbial diversity.

**Figure 4:**
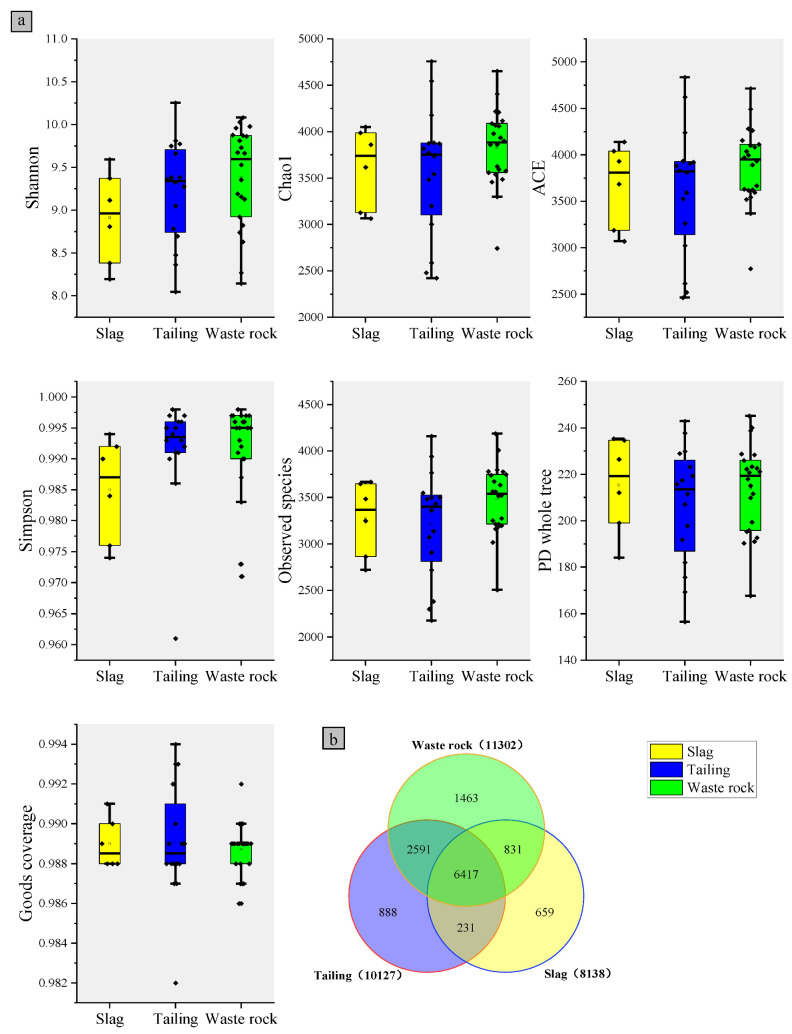
Rhizosphere microorganisms of three mine waste plants: (**a**) alpha diversity box plot and (**b**) OTUs number Venn plot.

Alpha diversity followed a similar pattern to OTU number, with M04 and M06 having the highest Shannon indexes, C04 and C06 having the highest Chao1 indexes, and S04 and S03 having the lowest Chao1 indexes (Table S2). The microbial α-diversity indices of the three mine waste types are shown in Figure 4a. Microbial diversity was highest in waste rock, followed by tailings, in which it was only slightly higher than in slag. A Venn diagram reflecting microbial OTU statistics in the rhizospheres of plants from the three waste types is shown in Figure 4b. As with α-diversity, waste rock had the highest OTU number, followed by tailing, then slag. The same OTUs were found in high abundance in waste rock and tailing areas, indicating that microbial community compositions were very similar in those two domains, whereas the OTUs in slag differed.

**Figure 5:**
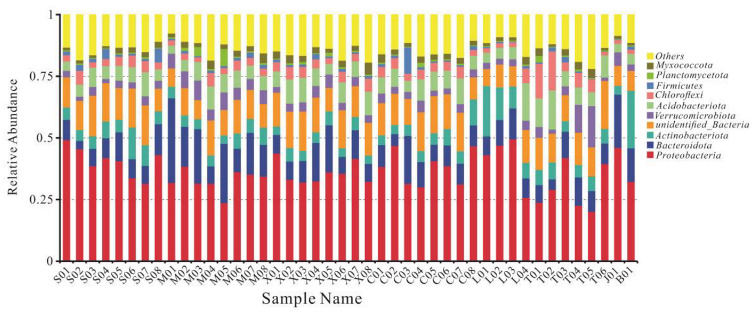
Relative abundances of phyla present in samples.

**Figure 6:**
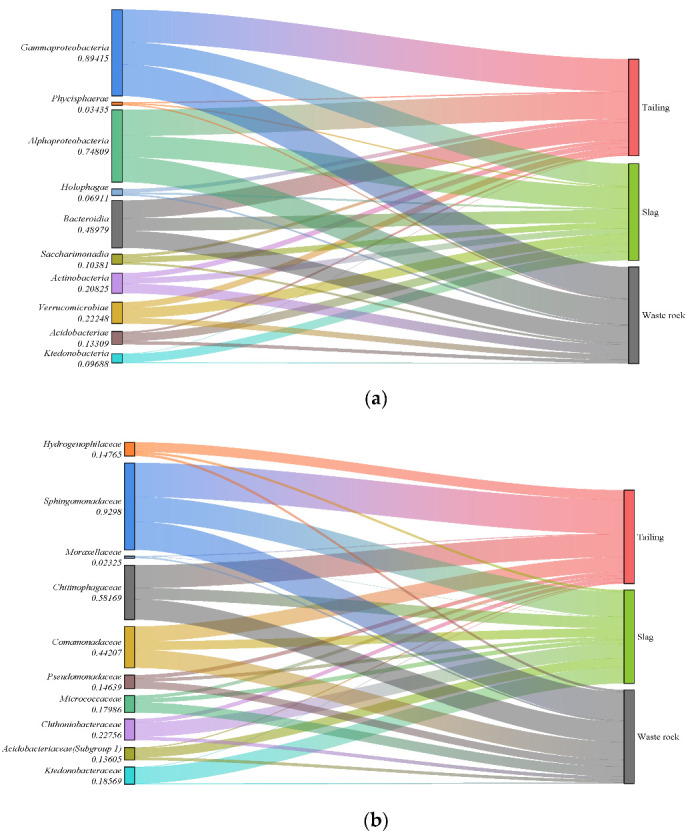
Sankey diagram of the composition of the rhizosphere microbial communities of three mine waste plants (only the top ten microorganisms in relative abundance are listed: (**a**): Class level; (**b**): Family level).

Sequences were taxonomically assigned to 90 bacterial phyla, 189 classes, 413 orders, 560 families, and 966 genera. With the exception of samples M01 and M05, the most abundant phylum in all samples were *Proteobacteria*, and the relative abundance of *Proteobacteria* in different samples ranged from 20 to 50% (mean 36%), followed by *Bacteroidota* (11%), *Acidobacteriota* (7%), and *Actinobacteriota* (6%) (Figure 5). The community composition also varied among different mine waste types. Sankey diagrams of the 10 most abundant microorganisms in each waste type are shown in Figure 6a. *Gammaproteobacteria* was the most abundant microorganism in tailing, *Actinobacteria* was mainly distributed in waste rock, and *Saccharimonadia*, *Acidobacteriae*, and *Ktedonobacteria* were mainly present in slag, which is consistent with a study reported by Wang et al. [55]. At the family level, the species compositions of the different waste types were more variable (Figure 6b). The vast majority of *Hydrogenophilaceae* were found in tailing, *Ktedonobacteraceae*, *Chthoniobacteraceae*, and *Acidobacteriaceae* (Subgroup 1) were mostly found in slag, and *Pseudomonadaceae* and *Micrococcaceae* were mainly found in waste rock, with a relative abundance up to twice that of tailing and slag.

**Figure 7:**
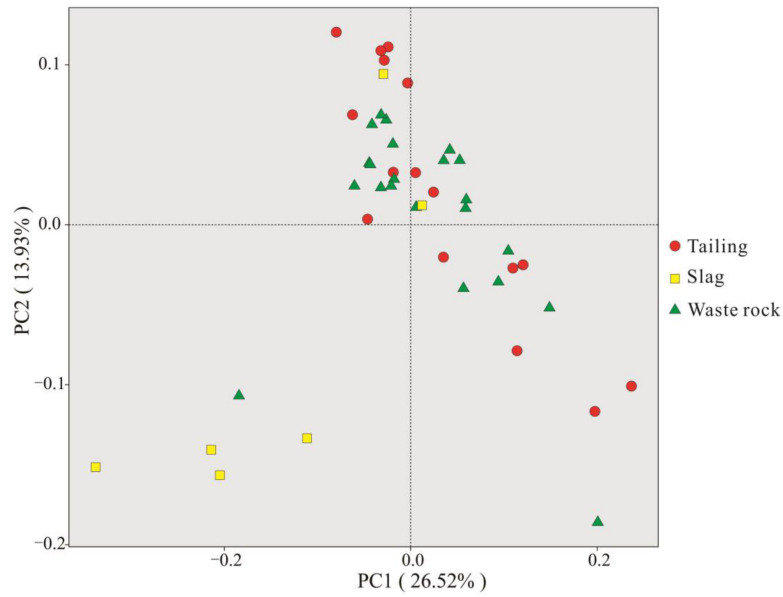
Analysis of the principal coordinates of the rhizosphere microbial OTUs of three mine waste plants.

The microbial OTUs for PCoA analysis are shown in Figure 7. The communities in tailing and waste rock areas were not very differentiated, but were clearly distinguished from those in slag areas, which is concordant with the Venn diagram.

### 3.3. Relationship between Geochemical Parameters and Microbial Communities

**Figure 8:**
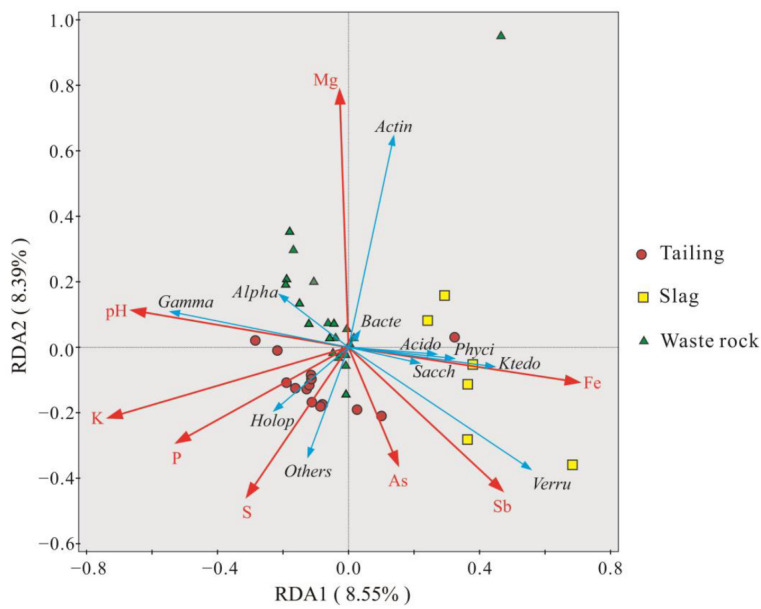
Redundancy analysis of the composition and geochemical parameters of the rhizosphere microbial community (Class level) of three mine waste plants. (Meaning of abbreviations in the figure: *Bacte*: *Bacteroidia*, *Gamma*: *Gammaproteobacteria*, *Alpha*: *Alphaproteobacteria*, *Actin*: *Actinobacteria*, *Verru*: *Verrucomicrobiae*, *Ktedo*: *Ktedonobacteria*, *Acido*: *Acidobacteriae*, *Sacch*: *Saccharimonadia*, *Phyci*: *Phycisphaerae*, *Holop*: *Holophagae*).

The compositions of plant rhizosphere microbial communities in the three mine waste types varied widely, which may be due to the combination of Sb contamination levels and other geochemical parameters. Redundancy analysis was performed on geochemical parameters at the class level to investigate interrelationships between geochemical parameters and microbial communities, and the results are shown in Figure 8. Two axes explained 8.55% and 8.39% of the variance. pH was positively correlated with *Gammaproteobacteria* and negatively correlated with *Ktedonobacteria*, *Phycisphaerae*, *Acidobacteriae*, and *Saccharimonadia*. Mg content was positively correlated with *Actinobacteria*. Compared with other geochemical parameters, the effect of As on microbial communities was relatively small. Sb and As content were positively correlated with the microbial communities in the majority of samples from tailing and slag, but they were negatively correlated with the microbial communities in waste rock. Mg had a significant effect on the microbial community in waste rock. Sb had a greater effect on the microbial community in tailing and slag.

## 4. Discussion

The geochemical parameters show that, after beneficiation and smelting, the Qinglong Sb mine waste is at great risk of contamination, and from another point of view, the Qinglong Sb mine contains considerable potential resources. The Sb and As content in slag and tailing was extremely high. Very high Sb content may be one of the reasons why there were few types of plants growing in slag. The smelting process causes the loss of As and S (sulfur) in slag and relative enrichment of Fe.

In general, high concentrations of heavy metal(loid)s are toxic to microorganisms and inhibit their growth, which in turn affects microbial communities and microbial diversity. In comparisons of the relative abundance of microbial communities and α-diversity in the three mine waste types, the microbial diversity in waste rock with relatively low Sb and As content was higher than that of the other two waste types, and it was hypothesized that the two metalloids had an inhibitory effect on the development of microbial communities. The main microorganisms in the mine waste were *Proteobacteria*, *Bacteroidota*, *Acidobacteriota*, and *Actinobacteriota*, which is consistent with previous studies [34,56,57,58,59].

*Proteobacteria* are the major Sb-resistant bacteria in the microbial community [60,61,62], and 99 of 125 culturable Sb(III)/Cu(II)-resistant bacteria from 11 different types of mining waste are Proteobacterial species, including α-*Proteobacteria* (mainly *Brevundimonas*) and γ-*Proteobacteria* (mainly *Pseudomonas*). *Actinobacteria* (mainly *Arthrobacter*) and *Firmicutes* are also considered to be highly resistant to Sb(III) [63]. *Pseudomonas* reportedly has the ability to dissolve heavy metals and form complexes with them, and they can resist heavy metals through bioaccumulation, and fix them via metabolic processes [64]. In addition to resistance to Sb, *Proteobacteria*, *Actinobacteriota*, *Firmicutes*, *Pseudomonadales*, *Comamonadaceae*, *Acinetobacter*, *Arthrobacter*, *Bacillus*, and *Hydrogenophaga* are among the species that have been reported to function in the oxidation of Sb(III) or to be involved in the biotransformation of Sb [61,65,66,67]. All these communities were present in considerable abundance in the samples tested in the current study (Table S3).

Compared with previous studies [68,69,70], the abundance of *Actinobacteriota* and *Arthrobacter* in plant rhizosphere microorganisms in antimony mining areas is significantly higher than that in non-mining areas. In the study of Sun et al. [71], the members of *Actinobacteriota* are considered to play a key role in the microbial community of active antimony tailing. Members of *Actinobacteriota* are usually identified as key groups in extremely oligotrophic environments. Oligotrophic bacteria are important contributors to various ecological cycles in nature. They are closely related to the cycles of nitrogen, carbon, sulfur, phosphorus, and trace elements in nature, and play a very important role in the self-purification process of environmental systems [72]. *Arthrobacter* is considered to have an important role in the ecological restoration of Pb–Zn tailings [73]. The study shows that *Arthrobacter* has the functions of phosphorus dissolution and nitrogen fixation [74]. These functions are essential for the growth of plants on oligotrophic mine waste. In addition, as an acidophilic and metal tolerant bacterium, *Arthrobacter* is considered to be an ideal plant growth-promoting bacterium (PGPB) in acidic mine waste [75]. Therefore, we suggest that *Actinobacteriota* and *Arthrobacter* are important taxa for reducing the heavy metal(loid) mobility, vegetation restoration, and self-sustaining ecosystem construction on antimony mine waste.

Redundancy analysis of microbial communities and geochemical factors indicated that Mg, Sb, and pH had the strongest effects on microbial community composition. pH has a strong influence on the structure and diversity of microbial communities. It can indirectly affect microbial community composition by changing the physical and chemical characteristics of the environment, for example by influencing the leaching of heavy metals [76,77]. In addition, pH directly affects the physiology and growth of microorganisms. When the pH value is neutral, microbial diversity is highest. When the pH value exceeds the survival range of one microorganism, most microorganisms will not survive [37,78]. The pH of tailing and waste rock was neutral to weakly alkaline, which is suitable for microbial development and promotes community diversity. Conversely, the acidic pH of slag reduces community diversity to an extent, and a similar situation has been found in other mining areas [79,80]. Some microorganisms were detected in the slag area, however, such as *Acidobacteriae* and *Ktedonobacteria*. In contrast, their abundance in tailing and waste rock was very low, indicating that these microorganisms can adapt to low-pH environments (Figure 6). In another study, *Acidobacteriae* was significantly enriched in acid soil that was severely polluted with heavy metals [81].

Sb was the main polluting element in the mining area, and its influence of it on the microbial community cannot be ignored. Studies have shown that Sb(III) can reduce the abundance of specific bacteria and bacterial diversity at the phylum level [82]. Adding Sb to the soil can significantly reduce substrate-induced resuscitation [83]. Sb can influence microbial diversity by affecting biological community-level physiological profile, and soil dehydrogenase activity [84]. In an Sb-polluted area, microbial composition and diversity were negatively correlated with Sb content [56]. Similarly, in the current study microbial diversity in slag—which had the highest Sb content—was lower than that in the other two types of mine waste.

**Figure 9:**
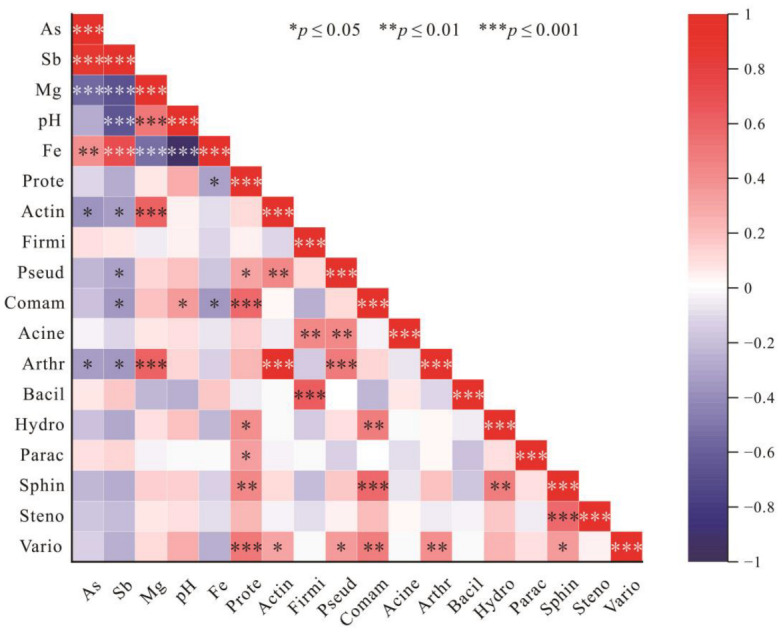
Heat map of Spearman correlation analysis between geochemical parameters and relative abundance of selected microorganisms. (Meaning of abbreviations in the figure: *Prote*: *Proteobacteria*, *Actin*: *Actinobacteriota*, *Firmi*: *Firmicutes*, *Pseud*: *Pseudomonadales*, *Comam*: *Comamonadaceae*, *Acine*: *Acinetobacter*, *Arthr*: *Arthrobacter*, *Bacil*: *Bacillus*, *Hydro*: *Hydrogenophaga*, *Parac*: *Paracoccus*, *Sphin*: *Sphingopyxis*, *Steno*: *Stenotrophomonas*, *Vario*: *Variovorax*.)

In previous studies, the abundance of some microorganisms resistant to As and Sb or involved in the biogeochemical cycling of As and Sb, such as *Actinobacteria*, *Firmicutes*, *Nitrospirae*, *Tenericutes*, and *Gemmatimonadetes*, was positively correlated with As and Sb content [85]. These microorganisms are considered to have functional genes and proteins related to As or Sb metabolism, including arsC, arrA, arsM, aioA, ArsB, and ACR3 [86]. In the present study, Spearman’s correlational analysis was performed by analyzing the relative abundance of several bacteria resistant to As and Sb or involved in As and Sb biogeochemical cycling processes such as *Proteobacteria*, *Actinobacteriota*, and *Pseudomonadales*, and the main geochemical parameters (Figure 9). As and Sb content were not significantly positively correlated with the abundance of these bacterial communities, and in fact the correlation was negative. Conversely, Mg was positively correlated with the abundance of several bacterial species, and it was highly correlated with the abundance of *Actinobacteriota* and *Arthrobacter*. Mg is an important component of some bacterial enzymes and plays an important role in stabilizing and regulating bacterial membrane structure and ribosomes [87,88,89]. It has also been shown that Mg has a crucial role in ATPase, and with increased Mg^2+^ concentration ATPase activity is enhanced [90]. ArsB, the transporter protein encoding Sb and As, is dependent on ATPase catalysis [64,91]. It is therefore hypothesized that the Mg deficiency in tailing and slag in the Sb mining areas has inhibited bacterial transport of Sb and As in vitro, resulting in reduced resistance to As and Sb. High levels of As and Sb are in turn harmful to bacteria, resulting in a negative correlation between As and Sb levels and the abundance of the corresponding bacterial communities.

## 5. Conclusions

In the current study, the Sb and As content in the three types of mine waste from the Qinglong Sb mine were very high. The investigation of plant rhizosphere microorganisms in the three types of mine waste indicated that there were significant differences in the microbial communities they contained. At the class level, the most abundant species in tailing is *Gammaproteobacteria*, *Actinobacteria* is mainly distributed in waste rocks, and the abundance of *Saccharimonadia*, *Acidobacteriae*, and *Ktedonobcteria* is the highest in slag. At the family level, *Hydrogenophilaceae* are enriched in tailings, and the abundance of *Ktedonobacterae*, *Chthoniobacacterae*, and *Acidobacteriaceae* (subgroup 1) in slag is significantly higher than that of the other two kinds of mine waste, while *Pseudomonadaceae* and *Micrococcaceae* were mainly found in waste rock. Since they are closely related to the cycling of nutrients such as nitrogen and phosphorus, *Actinobacteriota* and *Arthrobacter* are considered to be important groups in the oligotrophic mine waste environment. We suggest that *Actinobacteriota* and *Arthrobacter* can be used as important groups to reduce heavy metal(loid) mobility, revegetation, and create self-sustaining ecosystems in antimony mining areas. The microbial diversity in tailing was slightly higher than that in slag and waste rock is the highest. Increased Sb and As content reduced microbial diversity. Low pH reduces the diversity of rhizosphere microbial communities associated with slag plants, but it also promotes the development of some microorganisms in slag. The present study further revealed the composition and diversity of plant rhizosphere microbial communities in Sb mine waste. From the perspective of geochemistry, the regulatory mechanisms involved in microbial community compositions in Sb mine waste were further clarified.

## Figures and Tables

**Table 1:** Sample collection list.

Sampling Area	Plant Type	Sampling Quantity	Sample Number
Tailing area	*Trifolium repens* L.	4	S01–S04
	*Rumex acetosa* L.	4	M01–M04
	*Plantago asiatica* L.	4	C01–C04
	*Conyza canadensis* (L.) Cronq.	4	X01–X04
Slag area	*Polygonum capitatum*	6	T01–T06
Waste rock area	*Trifolium repens* L.	4	S05–S08
	*Rumex acetosa* L.	4	M05–M08
	*Plantago asiatica* L.	4	C05–C08
	*Conyza canadensis* (L.) Cronq.	4	X05–X08
	*Cynoglossumlanceolatum* Forssk.	4	L01–L04
	*Brassica juncea* (L.) Czern. et Coss.	1	J01
	*Raphanus sativus* L.	1	B01

## Data Availability

Raw sequences obtained in this study were submitted to NCBI Sequence Read Archive database under the accession number of PRJNA849367.

## Supplementary Materials

The following supporting information can be downloaded at: https://www.mdpi.com/article/10.3390/microorganisms10081507/s1, Table S1: The geochemical parameters of all samples; Table S2: Microbial α-diversity of all samples; Table S3: Relative abundance of microorganisms associated with biogeochemical cycles of Sb and As.
